# An Immunological Approach to Increase the Brain's Resilience to Insults

**DOI:** 10.1155/2014/103213

**Published:** 2014-04-24

**Authors:** En-Ju D. Lin, C. Wymond Symes, Andrea Townsend-Nicholson, Matthias Klugmann, Claudia B. Klugmann, Klaus Lehnert, Dahna Fong, Deborah Young, Matthew J. During

**Affiliations:** ^1^Functional Genomics and Translational Neuroscience Lab, Department of Molecular Medicine and Pathology, University of Auckland 85 Park Road, Grafton, Auckland 1142, New Zealand; ^2^Department of Molecular Virology, Immunology and Medical Genetics, The Ohio State University, BRT 912, 460 W 12th Avenue, Columbus, OH 43210, USA; ^3^Department of Biochemistry and Molecular Biology, University College London, Gower Street, London WC1E 6BT, UK; ^4^ViaLactia Biosciences (NZ) Ltd, UNISYS House, 650 Great South Road, Penrose, Auckland 1061, New Zealand

## Abstract

We have previously demonstrated the therapeutic potential of inducing a humoral response with autoantibodies to the *N*-methyl D-aspartate (NMDA) receptor using a genetic approach. In this study, we generated three recombinant proteins to different functional domains of the NMDA receptor, which is implicated in mediating brain tolerance, specifically NR1[21–375], NR1[313–619], NR1[654–800], and an intracellular scaffolding protein, Homer1a, with a similar anatomical expression pattern. All peptides showed similar antigenicity and antibody titers following systemic vaccination, and all animals thrived. Two months following vaccination, rats were administered the potent neurotoxin, kainic acid. NR1[21–375] animals showed an antiepileptic phenotype but no neuroprotection. Remarkably, despite ineffective antiepileptic activity, 6 of 7 seizing NR1[654–800] rats showed absolutely no injury with only minimal changes in the remaining animal, whereas the majority of persistently seizing rats in the other groups showed moderate to severe hippocampal injury. CREB, BDNF, and HSP70, proteins associated with preconditioning, were selectively upregulated in the hippocampus of NR1[654–800] animals, consistent with the observed neuroprotective phenotype. These results identify NR1 epitopes important in conferring anticonvulsive and neuroprotective effects and support the concept of an immunological strategy to induce a chronic state of tolerance in the brain.

## 1. Introduction

The* N*-methyl d-aspartate (NMDA) receptors are one of the major subclasses of glutamatergic receptors that have critical roles in normal physiological processes such as neuronal proliferation [1], migration [2], and plasticity [3], as well as in pathological conditions, such as epilepsy and neurodegeneration [4, 5]. Following observations indicating NMDA receptor as an important mediator of glutamate-induced excitotoxicity [6] and that excessive NDMA receptor activation contributes to neuronal death in both acute trauma and chronic neurodegenerative diseases [7, 8], intense research was directed to develop NMDA receptor antagonists as neuroprotective agents. However, despite the wealth of preclinical studies demonstrating the therapeutic efficacy of NMDA receptor antagonists in various animal models of neurological disorders, most of these antagonists failed to produce positive results in clinical trials [9].

One of the reasons underlying the poor outcome of these clinical trials was the early termination and failure to reach therapeutic doses due to significant adverse effects by the antagonists [10]. As an alternative strategy, we previously designed a genetic vaccine approach to induce a humoral response with autoantibodies to the NMDA receptor [11]. These autoantibodies were hypothesized to circulate within the vascular system and not affect basal brain function (thereby limiting potential adverse effects), until, following a stroke or seizures with disruption of the blood-brain-barrier (BBB), they would cross into the brain, antagonize NMDA receptors, and attenuate injury. Epitope mapping of the vaccine sera suggested conserved epitopes in animals that showed antiepileptic and neuroprotective activity. These epitopes lie within extracellular domains of NR1 such as the S1 and S2 domains, as well as the NH_2_-terminal R1-R2 region. Both of these domains are highly conserved in the NMDA receptor subunits and are involved in agonist binding, with S1-S2 containing the glycine binding site in the NR1 subunit [12, 13] and R1-R2 the spermine and ifenprodil sites [14].

Here, we designed and generated three truncated NR1 recombinant proteins each containing distinct functional domains: NR1[21–375] encompassing the R1-R2 domain, NR1[313–619], spanning the preM1 region through to the channel pore TM2 region, and NR1[654–800] consisting of the extracellular loop between TM3 and TM4 and containing the S2 lobe. These peptides were used as immunogens and evaluated in a kainate model of hippocampal neurotoxicity. This study refines the immunization strategy by identifying the critical epitopes for vaccine production, which will aid the development of an alternative strategy for treating epilepsy.

## 2. Materials and Methods

### 2.1. Animals

Male Wistar rats were housed in an animal care facility under a 12 hr light-dark cycle with controlled humidity and temperature. Chow pellets and water were available* ad libitum*. All animal experiments were approved by the University of Auckland Animal Ethics Committee. General health and weights of the rats were monitored weekly.

### 2.2. Generation of Recombinant NR1 Proteins

For heterologous expression in bacteria, the regions encoding the amino terminal subdomain of NR1 (NR1[21–375]), preM1, and transmembrane domains TM1 and TM2 (NR1[313–619]) and the membrane-proximal portion linking TM3 and TM4 (NR1[654–800]) were PCR-amplified from the mouse NR1 cDNA [11]. The Homer1a coding region was amplified from whole rat brain cDNA. The NR1[21–375] and NR1[313–619] BamHI/EcoRI fragments were inserted into the BamHI/MunI sites of pET3dR (containing a modified multiple cloning site in pET3 (Novagen, Madison, WI; K. Lehnert, unpublished)). The NR1[654–800] and Homer1a BamHI/EcoRI fragments were inserted into the BamHI/EcoRI sites of pET3dR. The sequence of all expression plasmids was verified by DNA sequencing. The recombinant NR1- and Homer1a fragments were expressed in* Escherichia coli* BL21(DE3) according to standard protocols. Inclusion bodies containing the recombinant proteins were isolated from cell lysates, washed by sonication, and sedimented in 100 mM NaH_2_PO_4_, 10 mM Tris-Cl, pH 8.0. Purity and size of the recombinant proteins were assessed by SDS-PAGE.

### 2.3. Vaccinations

Washed inclusion bodies were solubilized and denatured in 8 M urea and dialysed against PBS. The precipitated proteins were diluted to 0.5 mg/mL, mixed with an equal volume of aluminum hydroxide adjuvant (Imject Alum; Pierce, Rockford, IL), and injected twice (i.p.) into rats which were 11-12 weeks of age, 0.2 mL per injection (total dose 0.1 mg). Two weeks later, the same dose was repeated in a single i.p. injection.

### 2.4. Blood Sampling

Blood was taken from all vaccinated rats at three time points: prior to vaccination, 7 weeks after vaccination, and at sacrifice. At the first two time points, 0.4-0.5 mL samples were taken. At sacrifice, 8-9 mL of blood was collected by intracardiac puncture. Serum was obtained following coagulation and centrifugation (12,000 g, 10 min, RT) and stored at −20°C.

### 2.5. ELISA Screening of Immune Sera

Aliquots of the antigen proteins were solubilized in 0.5% SDS and coated on 96-well MaxiSorp plates (Nunc, Roskilde, Denmark). After blocking, serum samples were applied to the plates in series dilution and incubated overnight at 4°C. Bound IgG was detected with peroxidase conjugated secondary antibody (Santa Cruz, Dallas, TX) and quantified at OD_450_ following addition of Turbo TMB-ELISA substrate (Pierce). Antibody titers were calculated by taking the inverse of the dilution at 50% saturation.

### 2.6. Whole Brain Membrane Isolation and Solubilization

One half of a freshly dissected rat brain was homogenized in 15 mL of 20 mM Tris-HCl, pH 8.0, containing protease inhibitors (mini Complete, Roche, Manheim, Germany) and centrifuged (800 g, 20 min, 4°C) to remove whole cells and cellular debris. Following recentrifugation (54,000 g, 1 hr, 4°C), the membrane pellet was washed and resuspended in solubilization buffer (20 mM Tris-HCl, 1% Triton X-100, 5 mM EGTA, 2 mM EDTA, 1 M NaCl, pH 8.0 containing protease inhibitors) and incubated for 2 hr at 4°C. Insoluble matter was pelleted (100,000 g, 30 min, 4°C) and the supernatant was assayed for protein content using Biorad Protein Assay substrate (Biorad, Hercules, CA).

### 2.7. Antigen Capture ELISA

96-well MaxiSorp plates were coated with monoclonal NR1 antibody (mAB363; Chemicon, Temecula, CA) at 0.5 *μ*g per well. Following blocking, freshly prepared solubilized whole brain membranes were applied overnight at 4°C (15 *μ*g per well). Sera were applied to the washed plates at 1 : 90 or 1 : 810 dilutions and incubated overnight at 4°C. As a control, affinity-purified polyclonal NR1 antibody (Chemicon AB1516) was applied at the same dilutions. Detection of bound antibody utilised peroxidase-conjugated secondary antibodies and TMB substrate, as for standard ELISA screening, and OD at 450 nm was determined.

### 2.8. Immunohistochemistry

Serum IgG was purified on immobilised Protein G as per manufacturer's instruction using ImmunoPure (G) IgG Isolation Kits (Pierce) and dialysed against PBS. Coronal hippocampal sections (35 *μ*m) were cut from a perfused (4% paraformaldehyde) naïve rat brain and prepared for immunohistochemistry as previously described [15]. Polyclonal NR1 antibody (Chemicon AB1516; 1 : 200) or vaccine sera IgG (100 *μ*g/mL) was applied overnight at RT. Bound IgG was detected with biotinylated anti-rabbit IgG or anti-rat IgG and ExtrAvidin peroxidase was stained with DAB substrate.

### 2.9. Kainic Acid-Induced Seizure Model

Nine to 12 weeks after immunization, rats received a single i.p. dose of kainic acid (KA, BioVectra, Charlottetown, Canada; 10 mg/kg). Seizure activity was monitored over a 90 min period and scored by a “blinded” observer using the following scale: 0: no response, 1: immobility and staring, 2: wet dog shakes (WDS), 3: facial clonus (such as mastication and head nodding), 4: forelimb clonus (unilateral or bilateral), and 5: rearing and falling with forelimb clonus. Four days later, the animals were sacrificed. Blood was taken for serum analysis, and brains were removed and frozen to –80°C.

### 2.10. Terminal Deoxynucleotidyl Transferase-Mediated Biotinylated-dATP Nick-End Labeling (TUNEL Staining)

Twenty *μ*m coronal hippocampal sections were fixed, washed, and equilibrated in terminal deoxynucleotidyl transferase (TdT) buffer (Invitrogen, Carlsbad, CA). Sections were then incubated for 1 hr at 37°C in TdT buffer containing 40 *μ*M biotin-14-dATP (Invitrogen) and 150 U/mL recombinant terminal deoxynucleotidyl transferase rTdT. This was followed by brief incubation (15 min, RT) in 2xSSC buffer (0.3 M NaCl, 30 mM Na citrate, pH 7.2) and a 10 min blocking step. Detection of bound dATP was achieved by treating the sections with ExtrAvidin peroxidase (Sigma, St. Louise, MO) and DAB substrate. Three equally spaced sections across the dorsal hippocampus were analyzed by a blinded experimenter, who counted the number of TUNEL-positive cells in the CA1 and CA3 regions (of both hemispheres) and assigned a grade based on the average of 3 sections (Table 2). Due to the larger group of naïve controls, a subset of the group (*n* = 7) that reached stage 4 or beyond (*n* = 11) was randomly selected for TUNEL staining. In addition, one rat from the NR1[21–375] group and one rat from NR1[313–619] group were not analyzed due to poor processing of the brain.

### 2.11. Immunoblot Analysis of Hippocampal Extracts

Groups of rats were vaccinated as described above (*n* = 5 per group) and sacrificed nine days after the boost injection. Brains were removed and the hippocampus was dissected. To obtain a crude lysate, tissue samples were sonicated in 10 mM Tris-HCl, 2 mM EDTA, pH 7.5, containing protease inhibitors (mini Complete, Roche). The lysates were centrifuged (800 g, 20 min, 4°C) and the supernatants were assayed for protein content. Aliquots were prepared for SDS-PAGE and stored –20°C.

Twenty *μ*g of protein per tissue lysate underwent separation by SDS-PAGE and was transferred to a nitrocellulose membrane (Amersham Biosciences, Buckingham, UK). After blocking and probing with primary antibody (see below), bound antibody was detected with secondary antibody conjugated to horseradish peroxidase (Santa Cruz) and a chemiluminescent substrate (ECL Detection System, Amersham Biosciences). Each membrane was probed a total of three times. Between probes, the membranes were washed and reblocked. Primary antibodies used included GAPDH (Abcam), PSD-95 (Chemicon), ERK1/2, CREB, BDNF (Santa Cruz), and HSP70 (Stressgen, Victoria, Canada). Chemiluminescent signals were captured on film (Hyperfilm ECL, Amersham Biosciences) and quantified using the quantity one image analysis system (Biorad). Ratios were calculated for each protein signal with respect to GAPDH.

### 2.12. Statistical Analysis

Chi square tests were used to analyze differences in the proportions of vaccinated animals to progress through the seizure stages and the proportions of rats with stage 4 and above seizures with minimal hippocampal cell death. Latencies to reach each stage and protein expression in immunoblot assays were analysed by one-way analysis of variance (ANOVA) followed by pairwise comparison with Student's *t*-test, with a significance level of *P* < 0.05.

## 3. Results

### 3.1. Production and Confirmation of Antigen-Specific NR1 Antibodies

**Figure 1:**
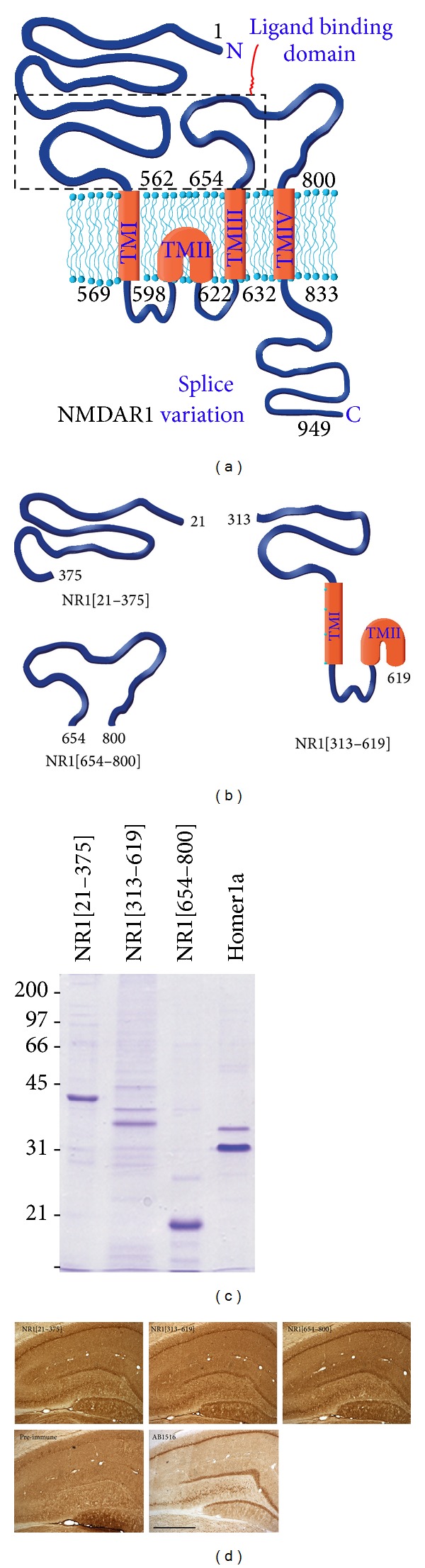
Generation of the NR1 fragments and screening of resultant vaccine antisera. (a) Schematic representation of the NMDAR1 subunit and (b) the engineered and generated NR1 fragments (figure adapted from http://www.pharmacology2000.com/Central/Opioids/Advanced_opioids3.htm). (c) SDS-PAGE analysis of washed inclusion bodies from* E. coli* expressing each of the NR1 fragments, showing degree of purity and molecular weight prior to vaccination. (d) Protein-G purified IgG from NR1 preimmune or immune rat sera was used at 100 mg/mL on naïve hippocampal sections. The affinity-purified commercial NR1 polyclonal antibody (Chemicon AB1516) was used at 1 : 200. Scale bar = 400 *μ*m.

NR1[21–375], NR1[313–619], and NR1[654–800] (Figure 1) were cloned into pET expression cassettes to generate recombinant protein with molecular size and purity assessed by SDS-PAGE (Figure 1(c)). Homer1a was chosen as a negative control antigen. All four proteins were used to vaccinate rats. ELISA screening of the sera against their respective antigens demonstrated a strong humoral response in all vaccinated rats that persisted for more than 4 months (Table 1(a)). Two complementary approaches were used to demonstrate that antigen-specific NR1 antibodies recognise and bind native NR1. Firstly, native NR1 from solubilized whole brain membrane was captured with immobilized NR1 monoclonal antibody and presented to immune sera in an antigen capture ELISA. OD_450_ signal showed that the three different NR1 antigens had generated antibodies with an affinity for the native protein with values for each serum correlating with its antigen-specific titer (Table 1(b)). The control Homer1a antisera did not recognise and bind to the captured native NR1. Secondly, IgGs from NR1[21–375], NR1[313–619], and NR1[654–800] rats were applied to naïve hippocampal sections with CA1, CA3, and dentate gyrus immunoreactivity identical to that of an affinity-purified commercial NR1 polyclonal antibody (Figure 1(d)). IgGs from preimmune rat serum did not bind to the hippocampus. Homer1a antisera recognized native Homer protein by immunoblot screening against crude brain extract (not shown).

### 3.2. Antiepileptic Effects of NR1[21–375] and NR1[654–800] Vaccination

**Figure 2:**
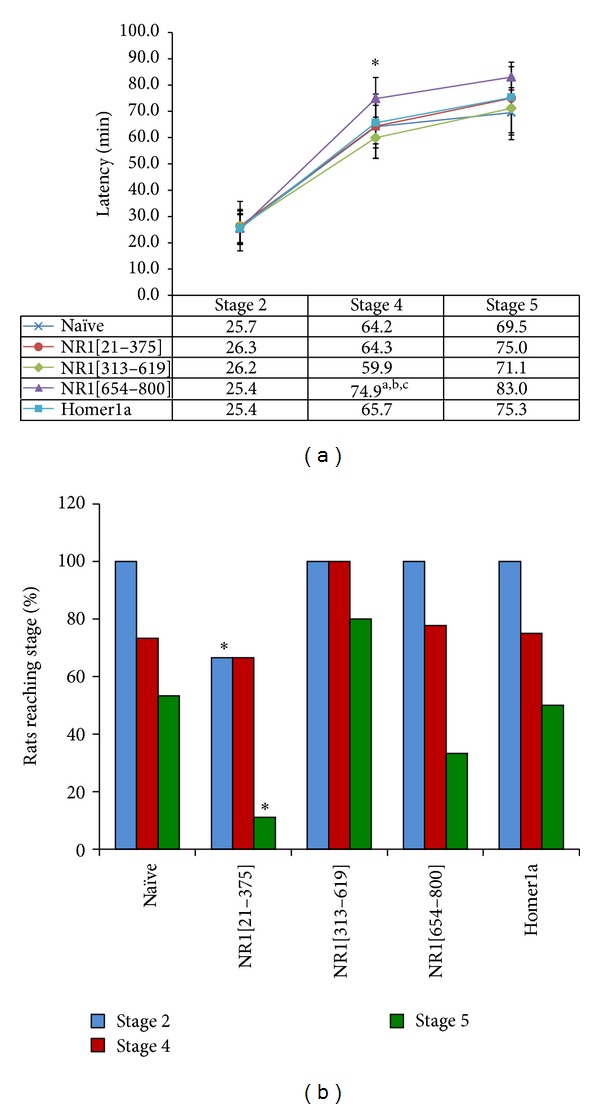
Effects of the NR1 fragments as vaccine antigens in a systemic KA seizure model. (a) Latency to reach each of the three defined seizure stages. Stage 2: wet dog shakes, Stage 4: forearm clonus, and Stage 5: forearm clonus accompanied by rearing and falling on back. Values represent mean ± SD. *P < 0.05, one-way ANOVA. ^a^ *P* < 0.05 with respect to naïve group, ^b^ *P* < 0.05 with respect to NR1[21–375], ^c^ *P* < 0.01 with respect to NR1[313–619]. (b) The percentage of rats to reach each of the stages. *P < 0.05, chi-square test.

At two to three months following vaccination, subgroups of male Wistar rats were administered kainic acid (KA) intraperitoneally to determine the anticonvulsive and neuroprotective efficacy of each vaccine antigen. In the naïve (nonvaccinated) group, systemic kainate induced a stereotypical epileptic phenotype. Of 15 naïve rats, 11 progressed to stage 4 behavioral seizures, and, of those, eight went on to stage 5 with mean latencies of 64.2 ± 8.0 and 69.5 ± 8.6 min, respectively (Figure 2(a)).

Notably, of nine NR1[21–375] rats, three did not develop any signs of seizure activity (*P* < 0.05 versus Homer1a and naïve controls) and only one progressed to stage 5 (*P* < 0.05 versus naïve control and *P* = 0.07 versus Homer1a) (Figure 2(b)). The NR1[654–800] group differed significantly from control groups with increased latencies to reach stage 4 seizures (one-way ANOVA *P* < 0.05, pairwise comparison, *P* < 0.05 versus naïve and *P* = 0.07 versus Homer1a). This group showed a moderate anticonvulsant phenotype with only three of nine rats progressing to stage 5 (Figure 2). The NR1[313–619] and Homer1a groups did not differ significantly from the naïve control group, with all mice exhibiting motor seizures and approximately half of each group progressing to stage 5 (six out of ten for NR1[313–619] and four out of eight for Homer1a).

### 3.3. NR1[654–800] Vaccination Protects against KA-Induced Cell Death

**Figure 3:**
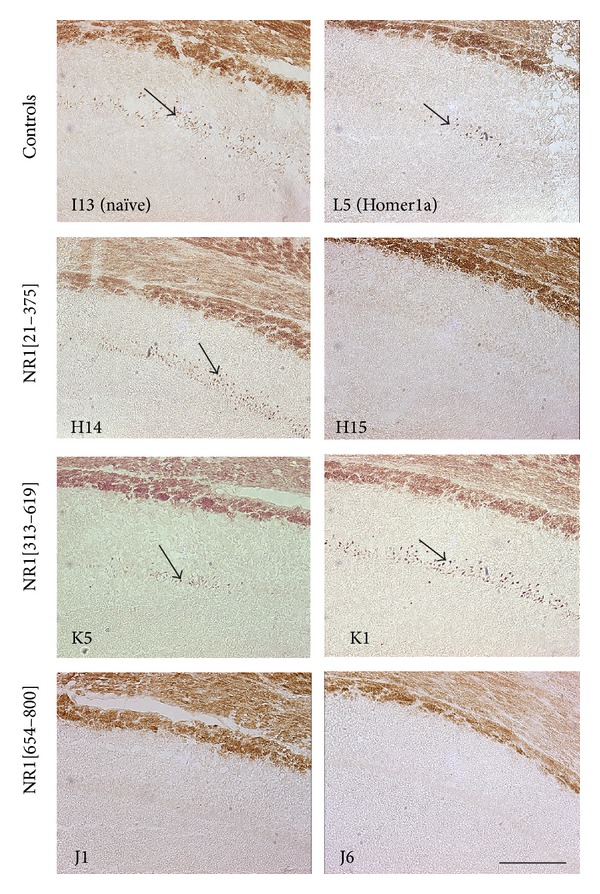
Seizure-induced damage in the hippocampus following systemic KA administration. TUNEL labeling (arrows) of neuronal cell death in the CA1 region of the hippocampus indicates the extent of damage in representative brain sections of NR1 vaccinated and control rats four days after receiving systemic kainic acid. Each image was from a different animal with the ID given in the bottom left corner. The vaccination treatment each rat had is given on the left. Scale bar = 200 *μ*m.

TUNEL staining of hippocampal sections of rats that developed stage 4 or higher behavioural seizures was analyzed at four days after systemic kainate administration. In naïve and Homer1a control rats, hippocampal neuronal cell death was observed in the majority of stage 4 or higher seizing rats (Figure 3 and Table 2). Despite significant anticonvulsive phenotype in NR1[21–375] vaccinated rats, kainate-induced damage in the subgroup that had stage 4 or 5 seizures was similar to that of naïve and Homer1a rats. Remarkably, in 6 of 7 NR1[654–800] rats that progressed to stage 4 and 5 seizures, neuronal damage was nonexistent, while the remaining one had minimal injury (*P* < 0.05 versus Homer1a or naïve groups). In contrast, moderate to severe tissue injury was seen in all but one of the NR1[313–619] vaccinated rats.

### 3.4. Selective Upregulation of Proteins Associated with Preconditioning in NR1[654–800] Vaccinated Rats

**Figure 4:**
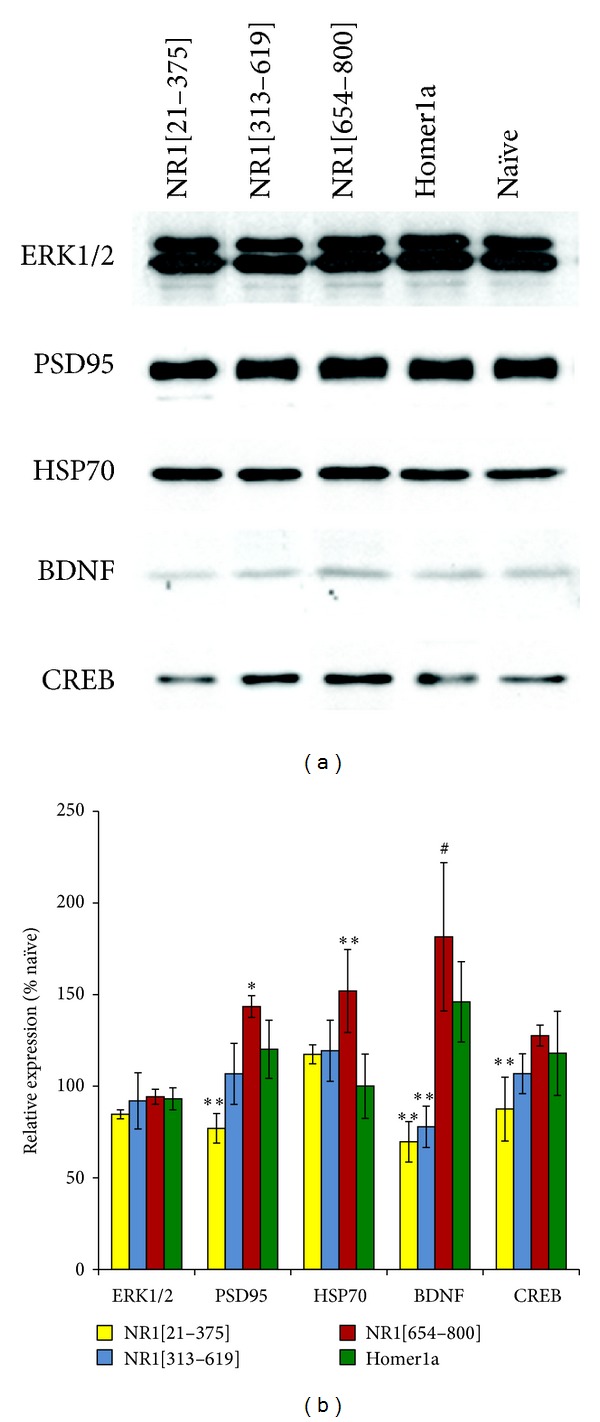
Expression analysis of hippocampal lysates from vaccinated rats. (a) Representative immunoblots of hippocampal samples obtained from vaccinated rats. For each group, samples from five individual animals were screened three times for every antigen, standardised to GAPDH, and graphed as a function of relative expression to a nonvaccinated group. (b) Values represent mean ± SEM. ^#^ *P* < 0.05, *P < 0.01, **P < 0.001 with respect to Homer1a rats.

Hippocampal lysates were prepared from additional rats and analyzed for expression of several proteins associated with the NMDA receptor and implicated in preconditioning (Figure 4). The NR1[654–800] group exhibited specific increases in protein expression of HSP70 (1.5-fold, *P* < 0.001 versus Homer1a and naïve controls) and BDNF (1.8-fold, *P* < 0.001 versus naïve and *P* < 0.05 versus Homer1a rats). Interestingly, there was a bidirectional change in the protein expression of PSD95, with a 23% reduction in the NR1[21–375] rats (*P* < 0.001 versus Homer1a and *P* < 0.006 versus naive rats) and a 43% increase in the NR1[654–800] rats (*P* < 0.005 versus Homer1a and *P* < 0.0001 versus naïve rats). In addition, the transcriptional factor CREB was slightly reduced in the NR1[21–375] rats (*P* < 0.004 versus Homer1a rats).

## 4. Discussion

In this study, we described an immunological approach that increases the brain's resilience to insults. Our results from systemic vaccination against NR1 peptide fragments are in line with our previous report showing strong antiepileptic and neuroprotective activity following peroral administration of AAV-NR1 [11]. We further identified two functional domains that have distinct neuroprotective effects. Systemic vaccination against the NR1[21–375] functional domain attenuated KA-induced seizures, whereas NR[654–800] vaccination was strongly neuroprotective against excitotoxicity-induced cell death.

Our original hypothesis for the neuroprotection induced by systemic NR1 vaccination was that cerebral insults enable IgG trafficking across a leaky BBB resulting in receptor binding, antagonism and neuroprotection. However, our data suggests that the efficacy of this vaccine did not result from acute BBB permeability and receptor blockade. First, we noted that NR1 IgGs are detectable in the CSF and are bound to antigen in the hippocampus in immunized animals under resting basal conditions [11]. Secondly, this study shows that marked changes in protein expression occur in the hippocampi of vaccinated animals prior to any insults. Our revised hypothesis is that sufficient NR1 IgGs pass an intact BBB to bind and allosterically alter a population of NMDA receptors, inducing a state akin to preconditioning. This would also explain the neuroprotective effect in immunized rats [11] as BBB would not be breached in animals not seizing following systemic kainite [16].

Preconditioning, the phenomenon that sublethal insults to cells induce a state of resistance to a subsequent stress, has been widely observed in organisms ranging from eukaryotes to prokaryotes [17–19], including man [20]. Both activation of NMDA receptors [21] and brief antagonism [22, 23] can induce a tolerance state. In models of ischemic preconditioning, tolerance was shown to be dependent on NMDA receptor activation and required new protein synthesis [24, 25]. Our revised mechanism is supported by increase in expression of proteins implicated in NMDA preconditioning such as BDNF and HSP70. The specific upregulation of the prosurvival factor BDNF in the NR1[654–800] immunized rats is in line with the observed neuroprotection in this group. Previous studies also suggested that NMDA and NMDA antagonist preconditioning exert their protective effect in part via stimulation of BDNF expression [23, 26, 27].

HSP70 is a molecular chaperone that is expressed constitutively and is induced by stressful conditions. Inducible HSPs prevent protein denaturation and improper polypeptide aggregation during exposure to physiochemical insults, thereby helping to maintain cellular integrity and viability [28]. There are extensive evidences for links between HSP70 overexpression and tolerance in preconditioning [29–31]. Blocking HSP70 inhibits the protective effects of thermal preconditioning against apoptosis and increases infarction volume after cerebral ischemia [31, 32]. The increase of HSP70 expression in NR1[654–800] immunized rats was observed nine days after the boost injection, more than three weeks after the initial vaccination, and without any physiochemical insults. This is distinct to traditional preconditioning responses, in which HSP70 upregulation is transient and usually diminishes within a few days [30, 33, 34]. The chronic preconditioned state induced by systemic vaccination described in this study thus represents a remarkable advance in utilizing the preconditioning phenomenon for neuroprotective strategy.

Our data suggests dissociation between seizure activity and cell death or survival as previously reported for several antiepileptic drugs (AEDs). For example, the NMDAR antagonist, MK-801, protects against kainate-induced neuronal damage without attenuating EEG seizures [35]. In a study of three AEDs in which two (PNU-151774E and diazepam) were strongly anticonvulsant, the third, lamotrigine, showed only a trend towards the prevention of seizures, yet all three protected susceptible neurons from kainate-induced cell death [36]. Despite lack of robust anticonvulsant activity in this model, lamotrigine is considered an efficacious AED, indicating the importance of neuroprotection alone in epilepsy drug development.

Although the mechanism underlying the distinct anticonvulsive and neuroprotective effects by NR1[21–375] and NR1[654–800] immunization is unclear, the bidirectional changes in PSD95 protein level observed may play a role and warrant further investigation. PSD95 proteins function as a scaffold to anchor NMDA receptors in the postsynaptic membrane and assemble a specific set of signalling proteins around them [37]. Levels of PSD95 protein may thus lead to differences in NMDA receptor signalling and its downstream effectors. The increase in hippocampal PSD95 in NR1[654–800] immunized rats that had enhanced neuroprotection against kainate seizure-induced cell death is consistent with the proposed mechanism for delayed neuronal ischemic preconditioning [38]. Neuroprotection in delayed ischemic preconditioning is mediated largely through the activation of NMDA receptors coupled to neuronal nitric oxide synthase (nNOS) via PSD95. The activation of nNOS pathway then leads to increased heat shock proteins and reduced expression of proapoptotic proteins. Based on the upregulation of PSD95, HSP70, and BDNF, it is likely that a similar mechanism underlies the neuroprotection observed in NR1[654–800] immunized rats. But a different mechanism is likely to account for the anticonvulsant effect observed in NR1[21–375] immunized rats.

Our data, in which we are proposing the therapeutic potential of inducing a humoral response to a specific domain of the NR1 subunit, seems paradoxical to the recent literature on anti-NMDA receptor encephalitis [39, 40]. However, the contrasting phenotypes observed in these two conditions suggest that there are distinct mechanisms in play. One potential explanation for this is the level of NMDA receptor antibodies present. The antibody titers reported for anti-NMDA receptor encephalitis patients, while they range greatly [39], are often two to three magnitudes higher than those reported in this study. Both neuroprotective and neurodestructive effects by NMDA receptors are well documented [4, 5]. It was proposed that responses to NMDA receptor activity follow a classical hormetic dose-response curve, meaning that both too much and too little can be harmful [5]. We propose that the modest increase in NR1 autoantibodies induced by systemic immunization triggered a subthreshold stress response leading to a preconditioned state that maintained NR1 expression and activity within an optimal state. In contrast, the massive production of NMDA receptor antibodies in anti-NMDA receptor encephalitis pushes the NMDA receptor activity to one end of the dose response curve, resulting in detrimental effects.

With NR1[654–800], we have developed an antigen with distinct prophylactic potential. This vaccine leads to long-term interaction of the induced NR1 autoantibodies with the receptor leading to cellular stress and adaptive responses akin to preconditioning as evidenced by chronic induction of HSP70. Although we do not fully understand how the IgGs alter NMDA receptor function, it is likely that they subtly alter the kinetics of the receptor sufficient to induce downstream signalling events. We have begun to identify some key targets in these signalling events, but the precise mechanism whereby the IgGs modulate NMDA receptors will be a focus of future studies. The phenomenon of preconditioning has significant potential as a neuroprotective treatment for many disorders. Preconditioning and tolerance have invariably been a relatively transient or acute phenomenon, therefore limiting the applicability for chronic and spontaneous, generally nonpredictable clinical events such as stroke, epilepsy, or trauma. The induction of circulating antibodies with the ability to continuously prime neurons prior to any insult removes this limitation, suggesting that immunization as a chronic preconditioning approach may have significant clinical potential.

## 5. Conclusions

In conclusion, we have identified that a functional domain of the NMDA receptor used as an antigen was remarkably neuroprotective. The same functional antigen which led to robust neuroprotection was selectively associated with a specific increase in expression of genes that are implicated in preconditioning including PSD95, BDNF, and HSP70. The other groups of vaccinated animals with characteristic hippocampal injury following kainate administration did not show an increase in these neuroprotective gene expressions. The study thus supports the concept of an immunological strategy to induce a chronic state of tolerance in the brain and may have implications for the treatment of conditions associated with neuronal death including epilepsy, stroke, trauma, and neurodegenerative disorders.

## Figures and Tables

**Table:** (a)

	Vaccination group			
	NR1[21–375]	NR1[313–619]	NR1[654–800]	Homer1a
Titer at week 7 (*n* = 20)	10082 ± 4884	10670 ± 4405	23457 ± 8300	27174 ± 14572
Titer at week 18 (*n* = 6)	5370 ± 1377	5712 ± 1471	8891 ± 1836	11586 ± 2886

Sera were collected 7 and 18 weeks after vaccination and antigen-specific titers were determined. Data are presented as mean ± SD.

**Table:** (b)

Vaccination group	Rat ID	Antigen-specific titer	OD at 450 nm	
			1 : 90	1 : 810
NR1[21–375]	H6	17556	0.432 (0.069)	0.185 (0.066)
	H3	8982	0.336 (0.066)	0.163 (0.069)
NR1[313–619]	K2	23879	0.491 (0.069)	0.484 (0.074)
	K19	13194	0.461 (0.079)	0.345 (0.058)
NR1[654–800]	J3	39149	0.516 (0.061)	0.364 (0.058)
	J15	23879	0.376 (0.057)	0.243 (0.065)
Homer1a	L6	41360	0.082 (0.062)	0.070 (0.062)
	L20	3729	0.068 (0.062)	0.066 (0.063)
AB1516			0.420	0.199

OD at 450 nm represents antibodies bound to immobilised NR1 protein in an antigen capture ELISA (see Section 2). OD at 450 nm for preimmune sera is shown in brackets.

**Table 2:** Seizure-induced hippocampal cell death.

Vaccination group	Number of rats examined	Hippocampal injury in CA1 and CA3			
		(−)	(+)	(++)	(+++)
naïve	7	2	2	1	2
NR1[21–375]	5	2	1	1	1
NR1[313–619]	6	1	0	2	3
NR1[654–800]	7	6*	1	0	0
Homer1a	6	2	1	2	1

The brains of rats to reach stage 4 or beyond were analysed by TUNEL labeling. Hippocampal injury was graded as follows: (−) no injury; (+) minimal injury (1–20 TUNEL labelled cells); (++) moderate (21–100 TUNEL cells); (+++) high (>100 TUNEL cells). *P < 0.05compared to either Homer1a or naïve groups.
